# Transporting results in an observational epidemiology setting: purposes, methods, and applied example

**DOI:** 10.3389/fepid.2024.1335241

**Published:** 2024-02-29

**Authors:** Ghislaine Scelo, Daniela Zugna, Maja Popovic, Katrine Strandberg-Larsen, Lorenzo Richiardi

**Affiliations:** ^1^ Department of Medical Sciences, University of Turin, CPO-Piemonte, Turin, Italy; ^2^ Section of Epidemiology, Department of Public Health, University of Copenhagen, Copenhagen, Denmark

**Keywords:** transportability, external validity, observational research, birth cohorts, TMLE

## Abstract

In the medical domain, substantial effort has been invested in generating internally valid estimates in experimental as well as observational studies, but limited effort has been made in testing generalizability, or external validity. Testing the external validity of scientific findings is nevertheless crucial for the application of knowledge across populations. In particular, transporting estimates obtained from observational studies requires the combination of methods for causal inference and methods to transport the effect estimates in order to minimize biases inherent to observational studies and to account for differences between the study and target populations. In this paper, the conceptual framework and assumptions behind transporting results from a population-based study population to a target population is described in an observational setting. An applied example to life-course epidemiology, where internal validity was constructed for illustrative purposes, is shown by using the targeted maximum likelihood estimator.

## 1 Introduction

Interest in external validity of study findings has increased in recent years, leading to a multiplication of theoretical publications in the field [see for example references (1, 2)], as well as raising concerns on how well target populations of health interventions are represented in study populations.

In 2002, Shadish et al. (3) defined external validity generalizations as “inferences about whether the causal relationship holds over variation in persons, settings, treatment, and measurement variables”. Lesko et al. (4) used a more technical definition referring on whether an internally valid effect obtained in a study is an unbiased estimator of the corresponding effect in a specific population of interest, further referred to as the target population. Depending on the nature of the target population, this definition may be consistent with the concepts of either generalizability or transportability: the former addresses the feasibility of applying a study finding to the background population from which the study population is a subset, the latter focuses on the setting where the study population is at least partly external to the target population (5). In observational analytical studies transportability is more relevant than generalizability as the intent is often to quantify the effect of an exposure on an outcome in contemporary populations to inform decision-makers. This is particularly true for life-course epidemiology, where long-term longitudinal data collection is in play. Under certain assumptions often not more stringent than those required for generalizability, study findings can be transported to different populations.

When a study is conducted with a specific target population in mind, the external validity of study findings could guide the choice of the sampling design. The gold standard would be a random sample of the target population, either through simple random sampling or other probability sampling methods that increase the study efficiency. This is however most often impractical because of the long latency that there might be between the exposures and their health effects, which requires to study the (far) past to guide current decisions when preventive or treatment actions should be implemented. Populations are “moving targets”, whose individuals are subject to dynamic processes in form of selection and self-selection forces, and thus, in the words of Keyes and Galea, “at any moment in time, the composition of a population is changing” (6). It follows that any study, representative and selected cohorts alike, is conducted in a selected population, which often will differ from the target population (7).

Most literature available in the field focuses on transporting study findings from randomized controlled trials (RCTs). Indeed, transportability requires internal validity in the first place and well-conducted RCTs may provide internally valid estimates of a causal relationship between the allocated treatments and an outcome. However, RCTs are relatively rare and often unethical in health-related research. Transporting study findings from observational studies is similarly feasible, assuming an internally valid estimate. Observational studies also confer the advantage of larger size, enhanced possibilities of long-term follow up and potentially higher external validity than RCTs considering that inclusion criteria are often more relaxed than in experimental settings. Westreich et al. (8) proposed the concept of target validity, which measures jointly the internal and the external validity of an effect estimate with respect to a pre-specified target population. In terms of target validity, a well-conducted observational study could outperform an RCT, as perfect internal validity does not translate into unbiased study finding in a specific target population.

Here we review the conceptual framework and assumptions behind transporting results from a study population to a target population in an observational setting. We detail one method through an applied example to life-course epidemiology where real data on covariates in the study and target populations were used to transport a simulated treatment-outcome effect from the study to the target population.

## 2 Assumptions

Transporting estimates from a study population to a target population requires a set of assumptions, some of which are verifiable whereas others are not (5). Careful consideration of assumptions listed below is needed in both cases. In Box 1 are provided the counterfactual framework notations for each assumption. For alignment with published literature that refers mainly to RCTs, “treatment” is used for any kind of exposure throughout this section.

Box 1 **Counterfactual framework notations and equations for the assumptions for transporting estimates from a study- to a target population.**

**Notations:**

• Outcome *Y* ∈ {0,1} (with *Y *= 1 indicating outcome occurs)
• Treatment *A* ∈ {0,1} (with *A *= 1 indicating treated)
• Baseline covariates *Z* (include effect modifiers)
• Study *S* (with *S *= 1 indicating selection into the study)
• Potential outcome *Y*^a^ under the treatment *A* = a

**Internal validity:**

1) Conditional treatment exchangeability: $Y^{\;a}⊥A\;|Z,\;S=1$ Mean (*E*) exchangeability over treatment:$\begin{array}{rl} & E(Y^{\;1\;-\;}Y^{\;0\;}|Z=z,\;A=a,\;S=1)\;=\;E(Y^{\;1\;-\;}Y^{\;0\;}|Z=z,\;S=1)\;\mathrm{for}\;\mathrm{every}\;a\;\in \;A \end{array}$
2) Positivity of treatment assignment: $\begin{array}{rl} & \mathrm{probability}\;P\;(A=a|Z=z,\;S=1)\;>\;0\;\mathrm{for}\;\mathrm{every}\;a\;\in \;A\;\mathrm{and}\;\mathrm{every}\;z\;\mathrm{with}\;\mathrm{positive}\;\mathrm{density}\;\mathrm{in}\;\mathrm{the}\;\mathrm{study}\;\mathrm{population} \end{array}$
3) No interference between subjects, and treatment definition consistency: $\mathrm{if}\;A\;=\;a\;\mathrm{then}\;Y\;=\;Y^{a}\;\mathrm{for}\;\mathrm{every}\;a\;\in \;A$

**External validity:**

1) Conditional exchangeability for study selection (generalizability assumption): Y⊥S|Zforeverya∈A Mean exchangeability of selection: $\begin{array}{rl} & E(Y^{\;1\;-\;}Y^{\;0\;}|Z=z,\;S=1)\;=\;E(Y^{\;1\;-\;}Y^{\;0\;}|Z=z)\;\;\mathrm{for}\;\mathrm{every}\;a\;\varepsilon \;A \end{array}$
2) Positivity of selection: $\begin{array}{rl} & \;\;\;\;\;\;P(S=1|Z=z)\;>\;0\;\mathrm{for}\;\mathrm{every}\;z\;\mathrm{with}\;\mathrm{positive}\;\mathrm{density}\;\mathrm{in}\;\mathrm{the}\;\mathrm{target}\;\mathrm{population} \end{array}$
3 and 4) No interference between subjects selected in the study vs. those not selected, and treatment version consistency between study population and target population: $if\;S\;=\;s\;\mathrm{and}\;A\;=\;a\;\mathrm{then}\;Y\;=\;Y^{a}\;\mathrm{for}\;\mathrm{every}\;a\;\in \;A$

### 2.1 Assumptions for internally valid estimates

1. Conditional treatment exchangeability: no unmeasured confounding of the treatment-outcome relationship in the study population. When estimating the population average treatment effect, it can be replaced by the weaker assumption of mean exchangeability over treatment, i.e., conditionally to the covariates, the distribution of the outcome, on average, differs among treated and untreated subjects due to treatment alone.
2. Positivity of treatment assignment: each subject in the study population has a positive probability of receiving each version of the treatment.
3. No interference between subjects: a subject's potential outcome is not affected by other subjects' exposure to the treatment.
4. Treatment definition consistency: well-defined treatment in the study population.

### 2.2 Assumptions for externally valid estimates

1. Conditional exchangeability for study selection: the outcomes are identical in subjects with equal treatment and covariate values in the study and target populations. For obvious reasons, the set of covariates cannot include those that separate the study population from the target population, such as geographical location or period when transporting from one context to another. When estimating the population average treatment effect, it can be replaced by the weaker assumption of mean exchangeability of selection, i.e., the distribution of the outcomes, on average, is equal in subjects with equal treatment and covariate values in the study and target populations.
2. Positivity of selection: sufficient overlap of the characteristics of subjects in the study population and the target population is a requirement. For example, if age is a covariate, transporting estimates from a study population with an age distribution very skewed compared to the target population will not be feasible. Large overlap will result in better precision of the estimates.
3. No interference between the subjects selected in the study vs. those not selected (i.e., there is no interference between individuals of the target population or between individuals of the study and the target population).
4. Treatment version consistency between study population and target population (i.e., the two populations do not have different versions of the same treatment)

Excluding the positivity assumptions, the assumptions listed in 1, 3, 4 are untestable. However, in some cases, sensitivity analyses can be carried out to evaluate the magnitude and direction of the bias under different scenarios of violation of these assumptions. Concerning the positivity of treatment assignment, it is possible to carry out basic descriptive analyses of treatment variability within strata defined by selected covariates. Similarly it is possible to analyse the overlap of the characteristics of subjects in the study population and the target population to check the positivity of selection.

While external validity can be jeopardized by several factors, including differences between the study and the target populations in subject characteristics, societal context, treatment/exposure (e.g., changed clinical practices/lifestyle), and outcome measurements (e.g., length of follow-up or timing of measurements), the assumptions listed above explicitly preclude variations other than in subject characteristics. Referring back to Shadish's definition of external validity generalizations as “inferences about whether the causal relationship holds over variation in persons, settings, treatment, and measurement variables” (3), the former variation in subject characteristics is the only one covered in currently available transporting methods. In other words, transportability methods assume that external validity bias arises solely from: (i) variation in the probability of being represented in the study population given certain subject characteristics; (ii) heterogeneity in treatment effects across subject characteristics, i.e., allowance of effect modification; and (iii) correlation between (i) and (ii).

## 3 Graphical representation

Exchangeability is a crucial assumption for both internal validity, i.e., applied to a specific study population, and external validity, i.e., applied to the comparison between the study and the target population. Directed acyclic graphs (DAGs) are a useful tool to visualize assumptions about exchangeability in the context of internal validity, using visual inspection and graphical rules. For a given set of relevant variables and an assumed data generating process depicted in a DAG, the exchangeability assumption is met when there are no open backdoor paths between the treatment and the outcome. We refer to the literature on DAGs for a summary of the graphical rules, known as d-separation (9, 10).

**Figure 1:**
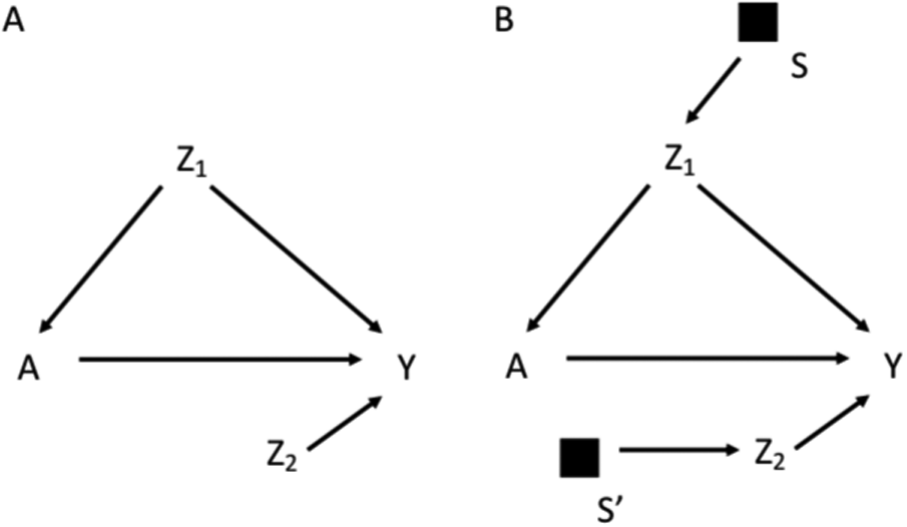
Directed acyclic graph depicting the effect of the treatment *A* on the outcome *Y*, in which *Z*_1_ is a confounder and *Z*_2_ is a determinant of *Y* (potential effect modifier) (**A**); selection diagram for transporting the effect of *A* on *Y* from a study population to a target population, which differs in the distribution of *Z*_1_ and *Z*_2_, as indicated by the *S*-variables (**B**).

Figure 1A for example reports the DAG for a target population, in which a treatment *A* affects the outcome *Y*, *Z*_1_ is a potential confounder of the effect of *A* on *Y* and *Z*_2_ is a determinant of *Y* (potential effect modifier). Given this DAG, to obtain a valid estimate of the causal effect of *A* on *Y* one could carry out the study in the target population and adjust for *Z*_1_ when estimating the association between *A* and *Y*. Adjustment for *Z*_1_ would block the only open backdoor path from *A* to *Y*. Pearl and Bareinboim (1, 11) defined as trivially transportable to the target population a causal relation that is identifiable in the target population, with no need of using information from the study population.

Most often, a study cannot be conducted on the target population, but it is possible to transport the estimates from a chosen study population. DAGs are insufficient to reason on the exchangeability assumption in the context of external validity, which depends on the difference between the target and the study populations, and on how this difference can modify the effect of treatment on outcome (11). External validity thus considers the structure of two distinct populations. For this reason, Pearl et al. in a series of studies have introduced the notion of selection diagrams, which are causal diagrams augmented with a set of variables that depict the mechanism underlying the relevant differences between the target and the study populations (11–14). For example, in Figure 1B the assumptions of different distributions of *Z*_1_ and *Z*_2_ between the study and the target population are depicted by the arrows pointing into *Z*_1_ and *Z*_2_ respectively. Following the notation introduced by Pearl et al., this difference is depicted by a set of variables S represented as black squares with arrows pointing towards *Z*_1_ and *Z*_2_. The S variables are dichotomous variables that can shift the distribution from the target population (*S* = 0) to the study population (*S* = 1), thus depicting the systematic processes, i.e., the mechanisms, accounting for the differences between the two populations. The absence of an *S* node pointing to a variable implies the strong assumption of invariance between the two populations for what regards the causal mechanism assigning the value of that variable. For example, in Figure 1B the distribution of *Y* differs between the two populations only as a consequence of the differences in the distributions of Z_1_ and Z_2_, as no S variable points directly to *Y*. The distribution of *A* also differs between the study and target population due to the different distribution of *Z*_1._

**Figure 2:**
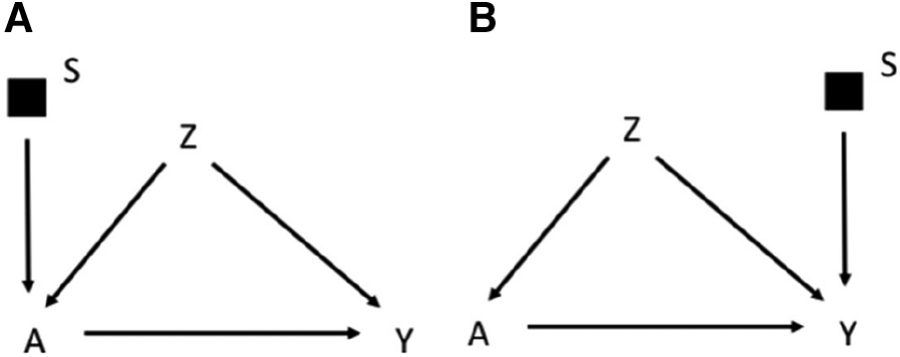
Selection diagram for transporting the effect of the treatment *A* on the outcome *Y* from a study population to a target population, which, as indicated by the *S*-variable, differs in the distribution of the treatment *A* (**A**) or the outcome *Y* (**B**).

The graphical rules that are used in DAGs to reason on exchangeability can be adapted to understand if and under what conditions an estimate is exchangeable in the context of external validity in selection diagrams. These rules are derived in a context in which the causal estimate in the study population is identifiable, i.e., internally valid, as it could be the case of an experimental study or an observational study with internal exchangeability between the exposed and the unexposed. A complete lack of *S* variables in a selection diagram (Figure 1A) implies that the causal estimate obtained in the study population is transportable to the target population with no need of calibration, as the two populations have the same underlying generating process and the same distribution of the variables (11). In presence of *S* variables, a causal estimate is transportable using recalibration techniques on the *Z* distributions if the open pathways from *S* to *Y* are blocked after conditioning on the *Z* variables (i.e., the *Z* variables d-separate *Y* from *S*) in a graph in which the incoming arrows to A have been removed. According to this rule the causal effect estimated in the study population is transportable in Figure 1B, if *Z*_2_ and *Z*_1_ are measured in the target population. If *S* and *Y* are d-separated without conditioning on *Z*, the estimate can be directly transported with no need of calibration on *Z* (11). It follows that an *S* variable that points directly into *A*, or has a connection with *Y* only through *A*, can be ignored, provided that the causal effect of *A* on *Y* can be estimated in the study population. An example of this scenario would be the selection diagram in Figure 2A, according to which there is no need to measure *Z* in the target population to transport the results of the study population. This is not an unusual scenario as often information on the target population comes from population registries that typically have more limited information on the potential confounders than the original study that provided the causal estimate. Conversely, in the diagram shown in Figure 2B, in which *S* affects *Y*, there is no guarantee that the effect of *A* on *Y* is transportable from the study population.

To summarize, depending on the selection diagram, the causal estimate identified in the study population could be (i) identifiable in the target population (trivial transportability) or (ii) transportable to the target population if, either directly (as in Figure 2A) or thanks to recalibration on the appropriate variables (as in Figure 1B, but not in Figure 2B), there are no open paths between *S* and *Y* in a graph in which incoming arrows to the exposure A are removed. These rules work in a non-parametric context in which any variable may act as an effect modifier, but they could be relaxed by assuming lack of interactions between the treatment and the potential effect modifiers. Furthermore, Pearl et al. (11) extended the approach to scenarios in which the S affects a treatment-dependent variable, which is a variable affected by the treatment, including, for example a mediator of the effect of the treatment on the outcome. For the sake of simplicity this scenario will not be discussed further in this manuscript.

## 4 Illustrative example

To illustrate the use of transporting estimates from a study population to a target population, we used data from an Italian birth cohort study (the NINFEA study, conducted in the Piedmont Region in 2005–2016) and transported a simulated exposure-outcome effect to the target population of newborns in the Piedmont Region in 2019. This setting allowed comparison with the same exposure-outcome effect directly estimated from the Piedmont Birth Register (PBR) data (since simulated), knowing that in a real-world setting the estimate would not be directly calculated in the target population.

### 4.1 Study population

NINFEA is a web-based birth cohort with the aim of investigating the effects of early-life exposures on the health of newborns, children, adolescents, and adults (15). Cohort members are children of women recruited between 2005 and 2016 in the Piedmont Region of Italy who completed a first online questionnaire at any time during their pregnancy on general health and exposures before and during pregnancy. Further follow-up information is obtained with repeated questionnaires when their child turns 6 months, 18 months, 4, 7, 10, 13, and 16 years of age. The study was approved by the local Ethical Committee (approval n. 45, and subsequent amendments). The NINFEA cohort includes 4,052 singleton pregnancies with residence in the Piedmont Region.

Previous work demonstrated that members of the NINFEA cohort study originate from a selected sample of pregnant women in Piedmont, with baseline participation strongly associated with socioeconomic factors, such as a high educational level (16). This baseline selection is not likely to affect internal validity (17), but may affect the external validity, as the participants of NINFEA were more often highly educated (64% vs. 30%), more likely to give birth for the first time (74% vs. 50%), and slightly older compared to PBR (Table 1). Thus, the difference in calendar period is not the only difference between the study and the target populations in our example.

**Table 1:** Observed distribution of covariates in the NINFEA cohort and the piedmont birth register (PBR) 2019.

	NINFEA (*N* = 4,052)	PBR (*N* = 26,909)
Maternal age (median, IQR)	33 (30, 36)	32 (28, 36)
Parity (*N*, %)		
0	2,994 (73.9)	13,332 (49.5)
1	868 (21.4)	9,869 (36.7)
2+	198 (4.9)	3,708 (13.8)
Maternal education (*N*, %)		
High	2,614 (64.5)	8,124 (30.2)
Medium	1,267 (31.3)	11,989 (44.5)
Low	171 (4.2)	6,796 (25.2)

### 4.2 Target population

A highly complete Birth Register exists in the Piedmont Region of Italy, which holds information on selected maternal and child/delivery characteristics on virtually all births in the region. To avoid potential recording delays due to the COVID-19 pandemic, we used anonymized data from 2019 (27,852 newborns, of whom 26,909 with complete data on the selected variables).

### 4.3 Exposure, outcome and covariates

The aim of the applied example is to estimate the risk difference of an outcome of interest between exposed vs. unexposed subjects in the target population, the PBR 2019, using data in the available study population, the NINFEA cohort. We simulated the treatment and the outcome, both binary, to guarantee that the causal estimate in the NINFEA cohort was internally valid, while we used real data for the covariates' distribution in the two populations. For the sake of simplicity, we selected three commonly used confounders/effect modifiers in birth cohort research of exposure-outcome associations: maternal age, parity, and education.

### 4.4 Statistical methods

Methodologies on how to transport estimates from the study to external target population are developing fast and there are pros and cons with the available methods (18–23). For the purpose of this illustration, we used the targeted maximum likelihood estimator (TMLE) method; other available methods are discussed in the next section. The TMLE method is a semiparametric double/multiple robust method with improved chances of correct model specification by allowing for flexible estimation using machine-learning algorithms (24, 25). Briefly, the average treatment effect (ATE) is defined as:

$$\begin{array}{rl} & \mathrm{ATE}=E(Y^{1}-Y^{0}|S=0)=\\ & \sum_{z}\{\left[E(Y|S=1,\;A=1,Z=z\right)-E(Y|S=1,A=0,Z=z)]\\ & *P(Z=z|S=0)\} \end{array}$$

where *Y* is the outcome, *A* is the exposure, *Z* is the covariates' vector and *S* is the sampling indicator indicating whether the sampling unit comes from the study population (*S* = 1) or the target population (*S* = 0). *Y*^a^ denotes the counterfactual outcome that would be observed if *A* = a, with a equal to 0 or 1. TMLE is solved in a iterative manner by initially finding *E*^0^(*Y*|*S* = 1,*Z*,*A*) using the independent sampling units from the study population and then updating the estimated conditional means *E*(*Y*|*S* = 1,*Z*,*A*) using consistent estimates of *P*(*S* = 1|*Z*) and *P*(*A* = 1|*S* = 1,*Z*) through the so-called clever covariates and the vector fluctuation parameter *ɛ*  = (*ɛ*_0_, *ɛ*_1_) (24):

$$\begin{array}{rl} E(Y|S=1,Z,A)(\varepsilon ) & =E^{0}(Y|S=1,Z,A)\\ & +\varepsilon_{0}\frac{\left(1-A)[1-P(S=1|Z)\right]}{P(S=1|Z)\;[1-P(A=1|S=1,Z)]}\\ & +\varepsilon_{1}\;\frac{A\;[1-P(S=1|Z)]}{P(S=1|Z)\;P(A=1|S=1,Z)} \end{array}$$

When there is little variability in *Y*-*E*^0^(*Y*|*S* = 1,*Z*,*A*), i.e., in absence of residual confounding, the fluctuation parameters *ɛ* will be estimated close to 0, meaning that *E*^0^(*Y*|*S* = 1,*Z*,*A*) was correctly specified. TMLE is doubly robust, i.e., it is consistent as long as either *E*(*Y*|*S* = 1,*Z*,*A*) or both *P*(*S* = 1|*Z*) and *P*(*A* = 1|*S* = 1,*Z*) are estimated correctly. As is evident from the estimation procedure, we need to know the complete individual data from the study population (i.e., *A*, *Z* and *Y*) but only the individual-level covariate data (i.e., *Z*) from the target population.

We considered the following data-generating mechanism which determines the exposure and the outcome assignments. Exposure assignment was generated for each subject *i* by sampling from *X*_i_∼*B*(π_i_) and the outcome assignment was generated by sampling from *Y*_i_∼*B*(*ρ*_i_) with π_i_ and *ρ*_i_ calculated as follows:

$$\begin{array}{rl} \pi_{i} & =\mathrm{plogis}[0.1*(\mathrm{ag}e_{i}-\mathrm{mean}(\mathrm{age}))-0.3*I(\mathrm{parit}y_{i}=1)\\ & -0.5*I(\mathrm{parit}y_{i}=2)] \end{array}$$

and

$$\begin{array}{rl} \rho_{i} & =\;\mathrm{plogis}[0.6*\;I(x_{i}=1)]+0.2*(\mathrm{ag}e_{i}-\mathrm{mean}(\mathrm{age}))\\ & -0.1*I(\mathrm{parit}y_{i}=1)-0.3*I(\mathrm{parit}y_{i}=2)+0.4*I(\mathrm{ed}u_{i}=\mathrm{medium})\\ & +0.6*I(\mathrm{ed}u_{i}=\mathrm{low})+0.3*\;I(x_{i}=1)*\;I(\mathrm{ed}u_{i}=\mathrm{medium})\\ & +0.5*\;I(x_{i}=1)*\;\;I(\mathrm{ed}u_{i}=\mathrm{low})] \end{array}$$

with plogis = exp(*v*)/(1 + exp(*v*)). Hence we identified maternal age and parity as confounders and maternal education as an effect modifier.

### 4.5 Results

**Figure 3:**
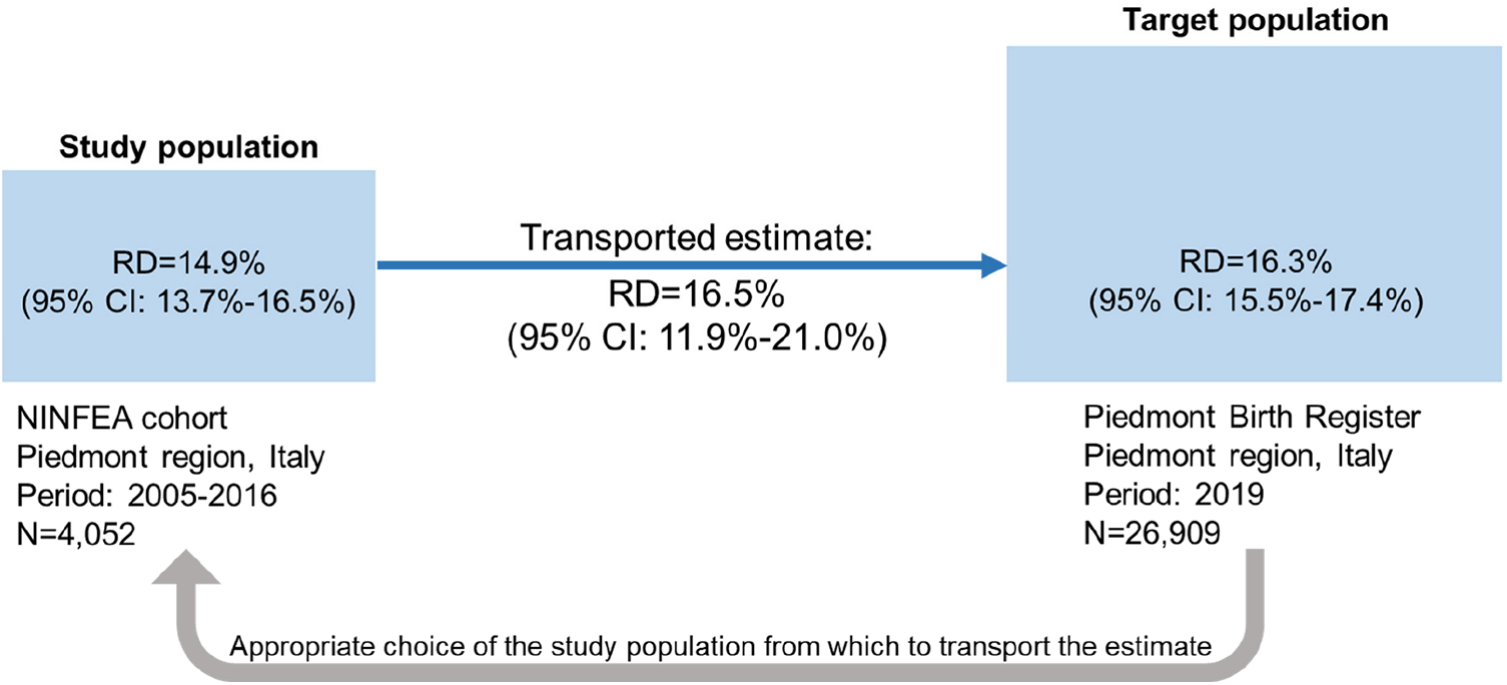
Illustration of transporting an exposure-outcome effect estimate from a study population (the NINFEA cohort study) to a target population (piedmont population in 2019) in presence of confounders and an effect modifier, which prevalences differ by populations. Ninety-five percent CI of the risk difference in the study and target populations is obtained by bootstrap, 95% CI of the transported risk difference is obtained by the sample variance of the estimated efficient influence curve.

The simulated exposure prevalence was 48.5% in the study population and 45.4% in the target population. The simulated outcome prevalence was 62.8% in the study population and 61.3% in the target population. Artificially the crude risk difference in both NINFEA and PBR populations was 24.4% (95% CI: 23.3%–25.7%). The adjusted observed marginal risk differences were 14.9% (95% CI: 13.7%–16.5%) in the NINFEA cohort and 16.3% (95% CI: 15.5%–17.4%) in the PBR data. When we transported the estimates from the NINFEA cohort into the PBR population using the TMLE method, the risk difference was equal to 16.5% (95% CI: 11.9%; 21.0%). In this example, the transported effect estimate is very close to the effect estimate in the PBR data (Figure 3).

## 5 Discussion

In this paper we reviewed the concept of transportability and showed an example of transporting the results from a birth cohort study to a target population of interest. A formal approach to transportability is an area of growing interest in epidemiology, mainly motivated by the need to apply the results of randomized controlled trials to specific populations of interest (2, 4, 8). The issue of external validity is however not limited to situation in which RCTs are feasible and were conducted (8, 11, 22). For example, in life-course epidemiology the evidence on the consequences of early-life exposures on later health outcomes is typically obtained from birth cohort studies and not from RCTs. We should then discuss not only if we can interpret causally the estimated associations (i.e., internal validity) but often also understand whether the available results can be used to support the decision-making in a specific target population (i.e., external validity).

Perhaps too schematically, when we need to obtain estimates in a given target population we should consider pros and cons of different options: (i) to run a study in a representative sample of the target population (ii) to identify the most valid estimate from a study carried out in a population that is nonoverlapping with the target and assume that it is applicable also to the target population without further reasoning on transportability issues, (iii) to formally transport the study estimates to the target population, which involves having access to the study and target population data. The first option is often unfeasible due to its costs, organizational complexities, and inherent very long lead times. It allows however a direct estimate of the estimand of interest (what Pearl et al. call trivial transportability, as described above), with no need of further assumptions but those used for internal validity. The second option is widespread, especially as decision making should be timely and often does not need an estimate of the exposure-outcome effect in the target population, but just relies on whether the exposure can cause the effect or not. Consistently, several frameworks have been suggested to assess causality in the context when multiple observational studies are available (26). This approach however does not estimate the population average treatment effect and cannot be used to assess the potential impact of a treatment in a population. The third option, which involves formal transportation of the study results to the target population is theoretically appealing but rarely used in practice, possibly because it involves strong unverifiable assumptions, methodological complexities, and requires access to some data of the target population.

This paper thus aims at promoting and facilitating the use of transportability methods in the context of observational research, and specifically life-course epidemiology. Note that the scenarios and the described methodology that we have introduced are far from being exhaustive. For example, Rudolph and van der Laan (23) analysed the TMLE performance under several scenarios when models are misspecified and the sampling positivity assumption is violated. Dong et al. (21) proposed a doubly-robust method based on an augmented estimator which combines an exposure, a sampling, and an outcome model to transport causal effect estimates from a study population to a target population. Josey et al. (22) have proposed calibration estimators to generate complementary balancing and sampling weights; this approach has strong parametric assumptions but, as opposed to the TMLE, allows to relax the propensity score exchangeability assumption, i.e., the hypothesis that the probability to receive the treatment given a set of measured covariates is the same in the target and study populations. Further we performed a complete-case analysis including only subjects with measured covariates vector in both the study and the target population. Simulation studies should be carried out to evaluate the performance of TMLE method, and more generally of different approaches, in transporting estimates from a study population to a target population, in particular when the missingness mechanisms involves both populations.

In conclusion, formally and quantitively transporting causal study estimates to a target population is an option that should be considered more systematically. This will involve continued methodological developments, widespread knowledge of transportability methods in the community of applied epidemiologists and closer collaboration between researchers of the original studies and investigators interested in specific target populations.

## Data Availability

Because the data are sensitive, synthetic data are available. Data on covariates have been obtained by randomly sampling with replacement from the original datasets, exposure and outcome have been simulated as described in the text. Data and R code for data simulation and TMLE implementation can be found at https://github.com/dzugna/transportability-tmle.git.
